# Multidrug-Resistant Moderate Tubercular Pleural Effusion: A Rare Case Presentation

**DOI:** 10.7759/cureus.56239

**Published:** 2024-03-15

**Authors:** Abhilasha Tiwari, Pankaj Wagh

**Affiliations:** 1 Respiratory Medicine, Jawaharlal Nehru Medical College, Wardha, IND

**Keywords:** multidrug-resistant, drug sensitivity, culturing, bedaquiline, pleural effusion, tuberculosis

## Abstract

Tuberculosis (TB) is among the most predominant infectious illnesses in developing areas around the globe. As stated by the World Health Organization (WHO), the number of instances of drug-resistant tuberculosis (DR-TB) has increased lately. This case report describes the effective diagnosis and customized treatment for primary extra-pulmonary multidrug-resistant tubercular pleural effusion, a disease which is difficult to identify due to relatively low bacterial count as well as frequently negative staining on Ziehl Neelsen (ZN) for acid-fast bacilli (AFB). The bacteria causing multidrug-resistant tuberculosis (MDR-TB) is resistant to a minimum of two drugs, isoniazid and rifampicin, the most effective TB medications. We are going to present the case of a 60-year-old male who complained of breathlessness, cough, and loss of weight for one month and chest pain and fever for 12 days. The patient's pleural fluid examination was carried out, which showed exudative fluid (according to Light's criteria) with adenosine deaminase (ADA) positive. Cartridge-based nucleic acid amplification test (CBNAAT) and line probe assays (LPAs) were carried out, which suggested *mycobacterium tuberculosis *(MTB)* *with rifampicin and isoniazid resistance. The patient was started an oral regimen with bedaquiline in accordance with WHO standards, leading to significant improvement. This case reveals that to promptly diagnose and treat DR-TB, pleural effusions, and pleural biopsies need to be exposed early to investigations such as Xpert (MTB)/resistance to rifampicin assay, culturing, and genotype drug sensitivity testing (DST).

## Introduction

The world's biggest infectious agent-related death cause is still tuberculosis (TB), even after 90 years of immunization and 60 years of medical therapy. *Mycobacterium tuberculosis *(MTB) is the obligate parasite that results in TB, a disease that mostly affects the lungs but can deteriorate any organ. It spreads between humans through the respiratory route [1]. Pulmonary tuberculosis (PTB) is the most prevalent clinical presentation of the disease, which accounts for 85% of the total recorded cases of TB worldwide. Extra-pulmonary tuberculosis (EPTB) is the term used to describe TB that affects any area of the body other than the respiratory system. Pleura is the most often affected anatomic location in EPTB [2]. It affects 5% of MTB-infected individuals. However, in regions with widespread TB prevalence, the frequency of PTB cases may reach as high as 30%. Diagnosing tuberculous pleural effusions is challenging as acid-fast bacilli (AFB) microscopy and culture commonly produce negative results in 48-96% of cases. More than 90% of patients have to undergo thoracocentesis, which is commonly used to detect a lymphocytic, exudative pleural effusion; in contrast, less than 10% of patients undergo direct examination to detect AFB [3]. We need alternative diagnostic approaches as AFB staining and culture usually yield negative results. Here, presenting a case, with primary extra-pulmonary MDR-TB with left-sided pleural effusion whose pleural fluid investigations were sent promptly, and resistance to rifampicin and isoniazid was revealed, which resulted in timely diagnostic strategy and management.

## Case presentation

**Figure 1:**
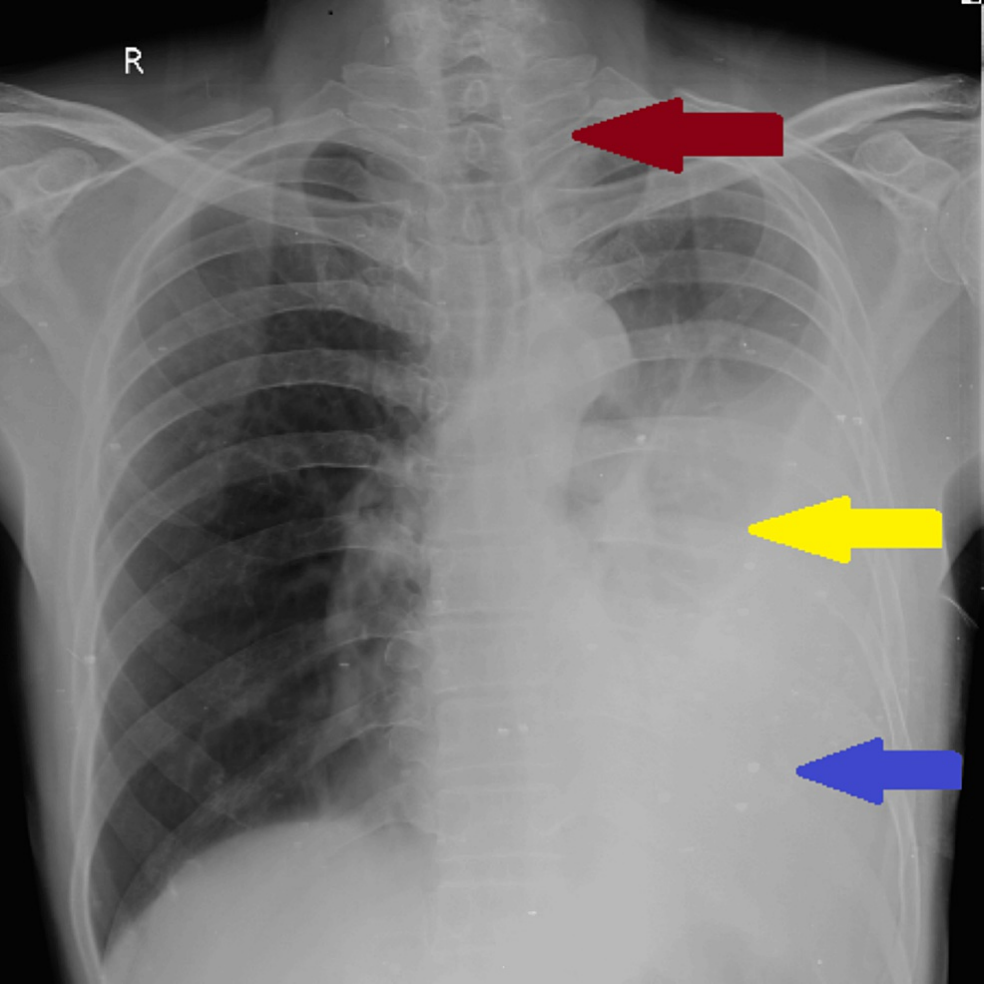
Chest X-ray PA view on admission showing left-sided pleural effusion. There is homogenous opacity on the left side of the chest (yellow arrow), shifting of the trachea to the right side (red arrow), and blunting of costophrenic and cardiophrenic angle PA, posteroanterior

A 60-year-old male was brought by relatives with chief complaints of breathlessness, cough, and loss of weight for one month and left-sided chest pain and fever for 12 days. The patient was apparently alright one month back when he developed breathlessness, which was insidious in onset and gradually progressive where he experienced breathlessness even during routine activities (Modified Medical Research Council grade IV). The patient also complains of a cough, which was insidious in onset and progressed over time, not associated with sputum production with no diurnal variation. The patient complained of pleuritic chest pain on the left side. He also had a fever with an evening rise of temperature, moderate grade, not associated with chills and rigors. The patient had a history of loss of appetite, generalized weakness, and loss of weight (more than 6 Kg a month). No history of abdominal pain, loose stools, pedal edema, hemoptysis, paroxysmal nocturnal dyspnea, or orthopnea. No history of diabetes, hypertension, PTB, ischemic heart disease, or bronchial asthma. No history of addiction and seasonal variation. Bowel bladder habits are normal. On examination, the patient was febrile with a temperature of 102°F, conscious, and oriented; pulse was 96 per minute, blood pressure was 120/70 mmHg, and oxygen saturation was 98%. The trachea appeared to be centralized. On percussion, a dull note was found on the left chest wall. Auscultation of the chest wall revealed bilateral vesicular breath sounds, which were decreased on the left chest wall. Chest X-ray posteroanterior (PA) view was performed for further assessment, which indicated a significant left-sided pleural effusion that was evident by the presence of homogenous opacity on the left side of the chest, tracheal shift to the right side, and blunting of costophrenic and cardiophrenic angle (Figure 1). 

An ultrasound-guided pigtail catheterization was carried out on the left hemithorax and the pleural fluid investigations were sent, the results so obtained are summarized in Table 1.

**Table 1:** Pleural investigations ADA, adenosine deaminase; LDH, lactate dehydrogenase

Investigations	Patient values	Reference values
Potassium	4.53 milliequivalents/L	3.5-5 milliequivalents/L
Serum lactate dehydrogenase	216 units/L	140-280 units/L
Pleural fluid LDH	450 units/L	>1000 units/L
Pleural fluid glucose	70 mg/dL	<60 mg/dL
Pleural fluid ADA	38.54 units/L	<40 units/L
Pleural fluid protein	4.78 g/dL	1-2 g/dL

**Figure 2:**
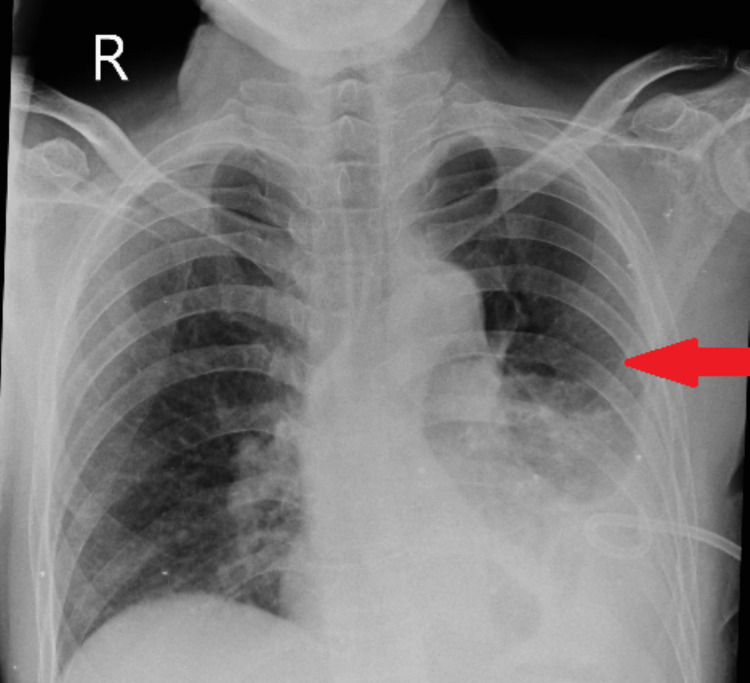
Chest X-ray PA view obtained following insertion of pigtail catheter on left side demonstrated a considerable clearance of pleural effusion (red arrow) after drainage of around 450 mL of pleural fluid PA, posteroanterior

A chest X-ray PA view was performed post-pigtail catheter insertion on the left side, which showed substantial resolution of pleural effusion (Figure 2).

Blood investigation showed normal hemoglobin, white blood cells, and platelet counts. His renal parameters, liver parameters, and electrolytes were within normal limits. Pleural fluid examination showed exudative fluid (according to Light's criteria) with adenosine deaminase (ADA) positive. CBNAAT showed MTB with rifampicin resistance. His pleural fluid cytology showed a cellular smear showing marked lymphocytosis in a proteinaceous background. There was occasional presence of degranulating neutrophils and mesothelial cells also. No malignant cell was seen, and AFB was negative on Ziehl Neelsen (ZN) staining. On gram staining eight to 10, low-power field pus cells were seen; less than 10, low power field epithelial cells were seen, and gram-positive cocci in group short chain scattered seen, gram-negative bacilli along with gram-positive bacilli were also seen. After that, the patient began an extensive oral MDR regimen. He was prescribed oral tablets bedaquiline 400 mg, linezolid 600 mg, clofazimine 100 mg, levoflox 750 mg, cycloserine 500 mg, pyridoxine 100 mg, pantoprazole 40 mg, acetaminophen, and tramadol hydrochloride. On follow-up, the patient showed improvement clinically and radiologically.

## Discussion

India holds the distinction of having the highest number of active TB cases globally. Over the past decade, there has been a concerning trend of increasing identification of DR-TB patients in the country. MDR-TB affects two to 3% of new infections and 12-17% of reinfection cases in India [4]. The National Tuberculosis Elimination Programme offers five types of DR-TB treatments: Isoniazid (H)-resistant TB, rifampicin (R)-resistant TB, MDR-TB (R and H resistant), pre-extensively DR-TB, and extensively drug-resistant [5]. Delayed diagnosis, insufficient drug supply, poor treatment, unsupervised therapy, substandard medication lack of education, and incorrect follow-up are among the factors that contribute to DR-TB [6]. Given the correlation between a positive HIV status and an elevated risk of contracting both drug-sensitive and DR-TB, it is crucial to screen for HIV infection in individuals diagnosed with EPTB [7]. There is very little data on primary multidrug-resistant pleural effusion; here we present a case of MDR EPTB highlighting the importance of early detection of drug resistance in EPTB. Without the initiation of a personalized anti-tubercular medication regimen based on each patient's unique drug resistance patterns, clinical and radiological betterment cannot be achieved. A comprehensive diagnostic approach, coupled with an appropriate medication regimen, in individuals with tubercular pleural effusion, can reduce the risk of significant pulmonary complications and ultimately reduce mortality and morbidity.

## Conclusions

For all patients with tubercular pleural effusion who are not responding well to the treatment, a pleural fluid study for drug resistance is necessary. The medication resistance assays need to be carried out in the main sample analysis in TB-endemic nations like India, more so because of its high prevalence in the country. As demonstrated by our case report, to prevent needless delays in diagnosis and early commencement of potent anti-tubercular treatment, it is advised that the Xpert® MTB/resistance to rifampin (RIF) test and LPA be performed as a first inquiry in all suspected instances of tubercular pleural effusion. Additionally, the pleural fluid should be tested for drug sensitivity testing (DST) to search for other drug resistance patterns to first- and second-line anti-tubercular medications. Therefore, maintaining a high level of suspicion is critical in cases of drug-resistant tubercular pleural effusion, especially in individuals who do respond to conventional anti-tubercular treatment regimens.
